# Financial and Psychosocial Distresses of Breast and Cervical Cancer Patients at the University College Hospital, Ibadan

**DOI:** 10.1200/GO.22.51000

**Published:** 2022-05-05

**Authors:** Damilola Busari, Mojisola Oluwasanu, Atara Ntekim, Prisca Adejumo, Funmilayo Olopade

**Affiliations:** ^1^ Department of Health Promotion & Education, Faculty of Public Health, University of Ibadan, Ibadan, Nigeria; ^2^ Department of Radiation Oncology, College of Medicine, University of Ibadan, Ibadan, Nigeria; ^3^ Department of Nursing, College of Medicine, University of Ibadan, Ibadan, Nigeria; ^4^ Center for Global Health, University of Chicago, Chicago, IL

## Abstract

The burden of Breast and Cervical cancer is increasing rapidly and these are leading causes of morbidity and mortality in women worldwide. The treatment and management of these illnesses are accompanied with both financial and psychological distresses to the patients. This study investigated the financial and psychosocial distresses of Breast and Cervical cancer patients at the University College Hospital, Ibadan, Nigeria.

**METHODS:**

This study was a descriptive cross-sectional study that employed a convergent parallel, mixed method design. The study was conducted among 105 breast and cervical cancer patients receiving treatment from the Radiotherapy clinic at the Radiation Oncology Department of the University College Hospital, Ibadan. Data was obtained using a semi-structured interviewer administered questionnaire and in-depth interview guide. The quantitative data was analyzed using descriptive and inferential statistics and qualitative data was analyzed using thematic analysis.

**RESULTS:**

The mean age of the respondents was 48.8 ± 10.0 years and almost half of the participants (45%) were aged between 51 and 60 years. Majority of the respondents (67.6%), were breast cancer patients while (32.4%) were cervical cancer patients. This study found out that (95.2%) of the respondents experienced high financial distress and (73.3%) of the respondents had a high level of psychosocial distress. There was an association between the level of financial distress of breast and cervical cancer patients and their psychosocial distress (*P* = .001). Factors that contributed to the financial and psychosocial distresses as deduced from the qualitative interviews were; high cost of treatment, loss of income and negative body image.

**CONCLUSION:**

This study revealed that breast and cervical cancer patients experience a high level of financial and psychosocial distresses and multi-component interventions such as health financing, advocacy, counseling, training and public enlightenment are proposed to address these challenges and improve quality of cancer care.

